# Impact of a drier Early–Mid-Holocene climate upon Amazonian forests

**DOI:** 10.1098/rstb.2007.0019

**Published:** 2008-02-11

**Authors:** Francis E Mayle, Mitchell J Power

**Affiliations:** School of GeoSciences, University of EdinburghDrummond Street, Edinburgh EH8 9XP, UK

**Keywords:** Amazon tropical forest, pollen, charcoal, fire, Holocene, climate

## Abstract

This paper uses a palaeoecological approach to examine the impact of drier climatic conditions of the Early–Mid-Holocene (*ca* 8000–4000 years ago) upon Amazonia's forests and their fire regimes. Palaeovegetation (pollen data) and palaeofire (charcoal) records are synthesized from 20 sites within the present tropical forest biome, and the underlying causes of any emergent patterns or changes are explored by reference to independent palaeoclimate data and present-day patterns of precipitation, forest cover and fire activity across Amazonia. During the Early–Mid-Holocene, Andean cloud forest taxa were replaced by lowland tree taxa as the cloud base rose while lowland ecotonal areas, which are presently covered by evergreen rainforest, were instead dominated by savannahs and/or semi-deciduous dry forests. Elsewhere in the Amazon Basin there is considerable spatial and temporal variation in patterns of vegetation disturbance and fire, which probably reflects the complex heterogeneous patterns in precipitation and seasonality across the basin, and the interactions between climate change, drought- and fire susceptibility of the forests, and Palaeo-Indian land use. Our analysis shows that the forest biome in most parts of Amazonia appears to have been remarkably resilient to climatic conditions significantly drier than those of today, despite widespread evidence of forest burning. Only in ecotonal areas is there evidence of biome replacement in the Holocene. From this palaeoecological perspective, we argue against the Amazon forest ‘dieback’ scenario simulated for the future.

## 1. Introduction

Understanding the direction and magnitude of climate change in Amazonia over the twenty-first century, and its impact upon Amazonian forests, constitutes a major international research effort that reflects the global importance of the Amazon forest biome and its associated climatic and hydrological systems (e.g. Malhi & Phillips 2004). The trend of rising temperatures in Amazonia (0.25°C per decade) measured over recent decades (Malhi & Wright 2004) is likely to continue, with a projected increase of 3.3°C this century under mid-range greenhouse gas emission scenarios, although this could be much higher (up to 8°C) under scenarios of widespread forest dieback (Betts *et al.* 2004; Christensen *et al.* 2007). However, trends of future precipitation change across Amazonia are much less clear, with the magnitude and direction of change depending on the climate model employed (e.g. whether or not ecosystem–climate feedbacks are included), the region of Amazonia considered and the timing of precipitation reduction (e.g. dry versus wet season; Bush & Silman 2004; Christensen *et al.* 2007; Malhi *et al.* 2008). The most alarming and controversial model result is the ‘Amazon dieback’ scenario by Cox *et al.* (2000) whereby positive feedbacks between increased forest dieback and increased aridity lead to a parched Amazon Basin completely denuded of forest by the end of the twenty-first century. This extreme scenario lies at one end of the range of hypothetical outcomes, the other being accelerated tree growth under conditions of enhanced fertilization due to higher CO_2_ levels (Lewis *et al.* 2004). Given the global implications of a deforested versus forested Amazon Basin, it is an urgent priority to better understand how its forests are likely to respond to drier climatic conditions.

Here, we address this issue by using a palaeoecological approach to examine how Amazonia's forests were affected by climatic conditions of the Early–Mid-Holocene (approx. 8000–4000 years BP (calendar years before present)) when major lake-level low-stands point to a significantly drier climate than today. We also consider how fire regimes (defined here as changes in charcoal abundance) may have changed throughout the Holocene, given that drier climates would be expected to promote increased fire, either directly due to drier soils and reduced humidity or indirectly by favouring more flammable ecosystems (e.g. savannahs). If past fire activity is found to be unrelated to climatic conditions or vegetation flammability, then this would be indicative of anthropogenic, rather than natural, burning.

## 2. Material and methods

**Figure 1:**
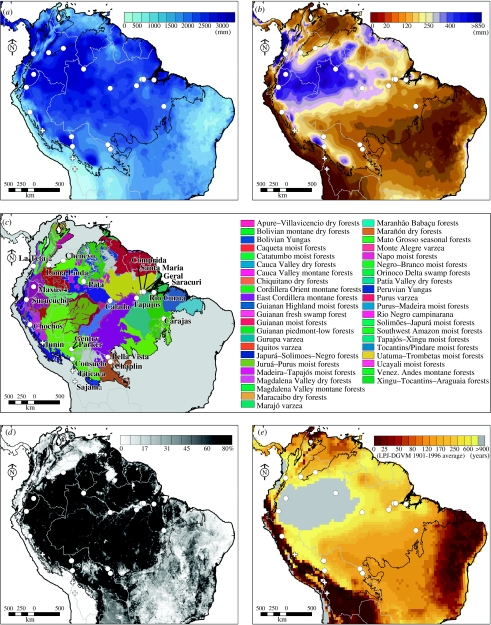
Maps showing site locations, distribution of forest types, tree cover, precipitation patterns and fire regime across the Amazon Basin. Amazonian forests are delimited by a solid black boundary line, which encompasses not only humid evergreen rainforests in the lowland basin and Guyana Shield but also semi-deciduous Chiquitano dry forests in the south and all forest types on the eastern flank of the Andes. WorldClim bioclimatic variables (Hijmans *et al.* 2005) of (*a*) annual precipitation and (*b*) precipitation of the driest quarter (driest three months) characterize present-day climatic variability across the Amazon. (*c*) Ecoregions (Olson *et al.* 2001) are shown for all forested areas. (*d*) The per cent tree cover map (Defries *et al.* 2000) illustrates the relative forest cover (available biomass) across the Amazon. (*e*) Simulated variations in historical (twentieth century) fire return intervals across the Amazon using LPJ-DGVM (Thonicke *et al.* 2001). Palaeovegetation and charcoal sites are shown by white circles; palaeoclimate sites are shown by white crosses.

**Figure 2:**
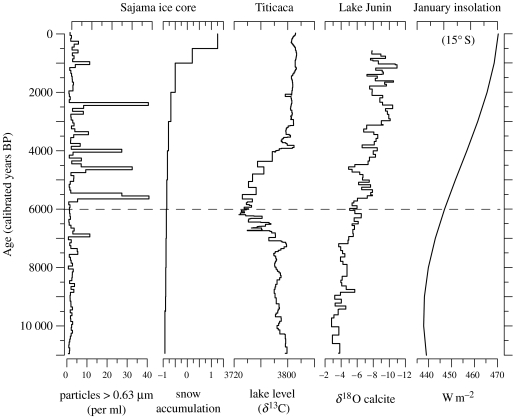
Palaeoprecipitation proxy data from selected sites from the tropical Andes. January insolation at 15° S latitude (Berger 1992) is shown in relation to: the Sajama ice core records (Thompson *et al.* 1998) of dust particles above 0.63 μm ml^−1^ and snow accumulation; lake-level changes as measured by δ^13^C at Lake Titicaca (Abbott *et al.* 2003); and Lake Junin precipitation inferred from δ^18^O of calcite (Seltzer *et al.* 2000).

**Figure 3:**
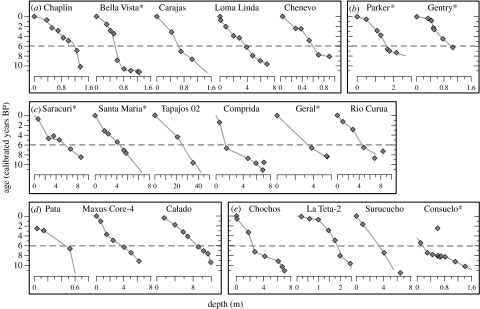
Age–depth curves for all sites discussed in the text ((*a*) lowland ecotonal sites, (*b*) southern sites, (*c*) eastern sites, (*d*) western and central sites and (*e*) Andean sites). Diamonds represent core-top samples and radiocarbon ages (calibrated years BP; Fairbanks *et al.* 2005). Asterisks denote sites where the authors' original age–depth model was used (solid line). For the remaining sites, age models were created by linear interpolation between dates. The dashed line indicates 6000 years BP, peak aridity at Lake Titicaca.

**Figure 4:**
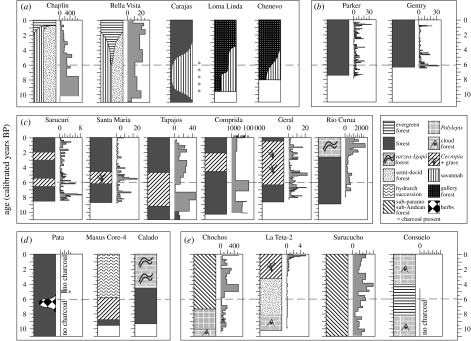
Palaeovegetation records, based upon previously published pollen data (see table 1 for site metadata), are presented in schematic cartoon form to illustrate the dominant changes (or lack thereof) in biome, forest type and/or species composition, in such a way as to illustrate the key vegetation responses and optimize inter-site comparisons. Site groupings are as follows: (*a*) lowland ecotonal sites, (*b*) southern sites, (*c*) eastern sites, (*d*) western and central sites and (*e*) Andean sites. Raw charcoal data are presented, which were obtained from the Global Charcoal Database (Power *et al.* in press; http://www.bridge.bris.ac.uk/projects/QUEST_IGBP_Global_Palaeofire_WG). Asterisks denote horizons where charcoal was recorded, but not quantified. Where no charcoal is shown for a site, this is because either charcoal was searched for but none was found (Consuelo and Pata) or charcoal was not searched for (Loma Linda, Chenevo, Maxus-4 and Calado).

We discuss a selection of previously published sites from tropical South America, which show strong evidence for precipitation change throughout the Holocene, all of which come from the tropical Andes (figures 1 and 2). We then synthesize previously published palaeovegetation (pollen data) and palaeofire (charcoal) records from sites across the Amazon Basin (including the eastern flank of the Andes) that occur within the present tropical forest biome (including ecotonal savannah gallery forests) and show their locations with respect to present-day forest types (ecoregions; Olson *et al.* 2001), tree cover (Defries *et al.* 2000), precipitation regimes (Hijmans *et al.* 2005) and fire return interval (Thonicke *et al.* 2001; figure 1). Only those sites with reliable chronologies and continuous records spanning most of the Holocene (figure 3) are considered. We use the authors' age–depth model from their published paper and, for sites without such a model, we produce our own based on linear interpolation between dates. Radiocarbon ages were calibrated into calendar years before present (BP) using the calibration curve by Fairbanks *et al.* (2005). The pollen records are summarized as simple schematic cartoons depicting significant biome shifts or compositional changes (figure 4). Raw charcoal data are presented alongside these vegetation records and are derived from the recently compiled, and publicly available, Global Charcoal Database (Power *et al.* in press; http://www.bridge.bris.ac.uk/projects/QUEST_IGBP_Global_Palaeofire_WG). Site metadata are shown in table 1.

## 3. Results and discussion

### (a) Holocene precipitation changes

There is widespread evidence that during the Early–Mid-Holocene (approx. 8000–4000 years BP) climatic conditions in the tropical Andes were significantly drier than present. Evidence comes from a variety of proxies (figure 2), e.g. peak dust concentrations and snow accumulation minima in Andean ice cores (Thompson *et al.* 1998), oxygen isotope ratios in lacustrine calcite (Seltzer *et al.* 2000) and, most convincingly, diatom, geochemical and seismic evidence for lake-level low-stands, particularly in Lake Titicaca (e.g. Baker *et al.* 2001) where lake-level dropped to 100 m below present between 6000 and 5000 years BP.

Given the complex spatial heterogeneity of precipitation patterns across Amazonia today (figure 1*a*,*b*), and the vast size of this region, it is unsurprising that the timing of these Holocene precipitation maxima and minima differs significantly among these records. Tropical Southern Hemisphere lake-level low-stands occur progressively later in the Holocene with increasing latitude (Abbott *et al.* 2003; Bush *et al.* 2005). For example, driest climatic conditions at Lake Junin (11° S), Lake Titicaca (14–17° S) and Sajama Mountain (18° S) were centred *ca* 10 000, 5500 and 4000 years BP, respectively (figure 2). This latitudinal, time-transgressive pattern points to the 20 000-year precession orbital cycle (Berger 1992) as the dominant driver of Holocene climate change (considered at multi-millennial scale), causing progressively greater austral summer insolation (and hence more southerly penetration of a stronger summer monsoon) throughout the Holocene.

Although the sites yielding these climate records occur in the tropical high Andes, they are relevant for this study because they receive most of their precipitation from the Amazon lowlands and, ultimately, the Atlantic Ocean (Nobre & Shukla 1996).

### (b) Impact of Holocene climate change upon Amazonian forests

#### (i) Lowland sites

The impact of the drier conditions of the Early–Mid-Holocene upon tropical forests varied across the Amazon Basin (figure 4), and, as expected, the magnitude of impact is inversely correlated with mean annual precipitation (figure 1*a*) and positively correlated with the length and severity of the dry season (figure 1*b*), and proximity to ecotones (figure 1*c*,*d*).

The greatest impact is evident at sites at the ecotonal margins of the basin where the dry season is longest and most severe (figure 1*b*). At Carajas (eastern Amazon ecotone), this reduction in precipitation caused replacement of forest by open savannah between *ca* 8900 and 4460 years BP, which in turn gave way to forest again when precipitation increased once more in the Late Holocene (figure 4; Absy *et al.* 1991; Sifeddine *et al.* 2001). Laguna Bella Vista and Laguna Chaplin lie at the southern Amazon ecotone within highly seasonal evergreen rainforest and are only 130 and 30 km, respectively, from semi-deciduous dry forests and savannahs to the south and east (figure 1*c*). Throughout most of the Holocene, these sites were dominated by a dry forest/savannah mosaic (figure 4; Mayle *et al.* 2000; Burbridge *et al.* 2004) when the climate was drier than today. Humid evergreen rainforests expanded to dominate the lake catchments only in the last two millennia, once precipitation had approached modern levels (reflected by rising water levels in Lake Titicaca following the Mid-Holocene low-stand). Similar biome shifts occurred at the northern Amazon ecotone, whereby gallery forests expanded within the Colombian llanos savannahs (Lagunas Loma Linda and Chenevo) (figure 4; Behling & Hooghiemstra 2000; Berrio *et al.* 2002*a*,*b*). However, the onset of forest expansion in response to increased precipitation at these northern sites (6910 and 7750 years BP, respectively) occurred significantly earlier than in southern Amazonia, consistent with the latitudinal time-transgressive precipitation changes identified in Andean lake-level records discussed earlier.

Interestingly, the Parker and Gentry sites of southern Amazonia (Bush *et al.* 2007*a*,*b*) are within 200 km of the Beni savannahs of northern Bolivia (figure 1*c*) and yet the surrounding tropical forests have experienced little change over the past 6000–7000 years (figure 4). However, this is perhaps unsurprising when one considers that these Peruvian forests receive more precipitation than those further south near the southern margin of Amazonia (figure 1*a*,*b*), and, crucially, the Beni savannahs are not climatically controlled but are instead a function of edaphic and hydrological conditions that are unfavourable to woody plants (Mayle *et al.* 2007).

The cluster of five sites in eastern Amazonia (Sarucuri, Santa Maria, Comprida, Geral and Tapajos; figure 1) all show signs of forest disturbance, revealed by substantial peaks in pollen (30–40%) of the weed tree *Cecropia* (figure 4; Bush *et al.* 2000, 2007*b*; Irion *et al.* 2006). This region presently experiences a significant dry season (figure 1*b*), which conceivably was longer and more severe earlier in the Holocene (if the Andean climate records are representative of the eastern Amazon). It is possible that increased severity and/or frequency of droughts led to greater tree mortality and hence more forest gaps, leading to a greater proportion of the forest in an early successional (i.e. *Cecropia*-dominated) state (Weng *et al.* 2002). However, as Weng *et al.* acknowledge, it is hard to envisage such a mechanism producing a *Cecropia*-dominated forest lasting several centuries or millennia, as one would expect that the short-lived, early pioneer, *Cecropia* trees would be rapidly out-competed by more drought-adapted (e.g. semi-deciduous) and longer-lived tree taxa. Furthermore, the marked variability in timing of this *Cecropia* phase among this tight cluster of sites does not fit with a regional climatic forcing and instead points to a non-climatic cause for this forest disturbance (e.g. humans) for at least some of the sites (see below).

Two riverine sites (Curuá and Calado), tributaries close to the main Amazon River channel (figure 1*c*), show a change from *terra firme* forests to predominantly *varzea–igapó* (seasonally flooded) forests in the Late Holocene (figure 4), consistent with higher flood levels of the Amazon River, although the extent to which this was caused by increasing precipitation and/or rising sea levels is unclear (Behling & da Costa 2000; Behling *et al.* 2001).

Lake Pata and Maxus-4 occur in the wettest part of the Amazon Basin (figure 1*a*,*b*), and each exhibits floristic changes during the Mid-Holocene, coincident with peak aridity in the high Andes. The Lake Pata pollen record has previously been interpreted by the site investigators (Colinvaux *et al.* 1996; Bush *et al.* 2002, 2004*a*) as indicative of a closed-canopy forest, throughout not only the Holocene, but also the last glacial–interglacial cycle. However, a close inspection of the Holocene sequence reveals a distinct pollen assemblage zone, centred *ca* 6000–7000 years BP, characterized by peaks in the herbs *Borreria* and Poaceae and the *Mauritia* palm, as well as the almost complete disappearance of Moraceae/Urticaceae pollen (figure 4; Bush *et al.* 2004*a*). Considered as a whole, this pollen assemblage is suggestive of a change from a closed-canopy forest to a forest/woodland sufficiently open to support a herbaceous understorey, consistent with a response to reduced precipitation. The latter is corroborated by geochemical evidence for lower lake levels at Lake Pata during the Early–Mid-Holocene (Bush *et al.* 2002). The most compelling evidence for such a vegetation change at this site comes from the negligible percentages of Moraceae/Urticaceae pollen, which are in marked contrast to the consistently high values of this pollen type (20–30%) not only throughout the rest of the Quaternary record of this site, but also in modern rainforest pollen samples elsewhere across the Amazon rainforest biome (e.g. Gosling *et al.* 2005). However, the very low sedimentation rate at Lake Pata means that the duration and timing of this Mid-Holocene vegetation zone is uncertain.

In contrast to Lake Pata, where *Cecropia* is absent *ca* 6600 years BP and never exceeds 10% anywhere in the record, the Ecuadorian site, Maxus-4, was dominated by *Cecropia* (approx. 60%) between 8700 and 5800 years BP (figure 4). The timing of this *Cecropia* peak, and the proximity of this site to the neighbouring Andes, raises the possibility that it might be due to episodic droughts under a drier climate causing increased gap formation, and thus favouring this pioneer species (Weng *et al.* 2002), although, as argued above, there are problems with this hypothesis, strengthening the case for other types of disturbance (e.g. burning by Palaeo-Indians) considered below.

#### (ii) Andean sites

Pollen records from the Andean flank of the Amazon Basin show how past drier conditions affected different kinds of forest in different ways. At Lago Consuelo, located in the lower montane cloud forest of Peru (1360 m elevation; figure 1*c*), reduced precipitation (9000–5000 years BP) caused local replacement of cloud forest taxa by lowland rainforest taxa (figure 4; Bush *et al.* 2004*b*). Further north in Peru, at the higher elevation site Laguna de Chochos (3285 m), cloud forest was replaced by *Polylepis* forest *ca* 10 000 years BP, which persisted until 7300 years BP, consistent with drier conditions (figure 4; Bush *et al.* 2005). In the Colombian Andes (Teta-2, 1020 m), the replacement of cloud forest by semi-deciduous dry forest *ca* 8000 years BP is also indicative of a change to a drier climate (figure 4; Berrio *et al.* 2002). In contrast, however, pollen from Lake Surucucho, located at the sub-paramo/sub-Andean forest ecotone in Ecuador (3180 m; figure 1*c*), shows surprisingly little change in forest composition throughout the Holocene (Colinvaux *et al.* 1997).

### (c) Holocene fire activity

Figure 4 demonstrates that there is considerable heterogeneity in fire regimes, both spatially and temporally. To what extent are these palaeofire records a function of climate, vegetation type and/or human activity? At Chaplin and Carajas, fire activity closely follows changing extent of flammable savannahs, demonstrating that past fires have long been a natural feature of savannahs in these ecotonal areas. Given that even drought-tolerant semi-deciduous dry forests are rarely subject to natural fire (as indicated by the presence of fire-intolerant cacti; Pennington *et al.* 2006), and some Holocene records are largely, or completely, devoid of charcoal (e.g. Pata and Consuelo), our working hypothesis is that the charcoal records from the remaining sites that have been forested throughout the Holocene reflect anthropogenic fires set, either intentionally or accidentally, by Palaeo-Indians. However, heterogeneity in the charcoal signals, even among densely clustered sites, suggests that such fires were localized and much smaller in scale than those of today (Bush *et al.* 2007*a*).

Despite this heterogeneity, there is a hint of a broader scale regional pattern, suggesting that climate forcing may also have played a role in Holocene fire regimes. Several sites (e.g. Santa Maria, Geral, Surucucho, Chochos) show clear Mid-Holocene (6000–4000 years BP) charcoal peaks, perhaps due to the drier climate at this time making forests more combustible. The absence of charcoal at Consuelo, and near-absence at Pata, is unsurprising, given that these sites occur in the wettest parts of the basin (figure 1*a*,*b*), although the presence of charcoal in a single sample at Pata *ca* 5000 years BP (M. Bush 2007, personal communication) raises the possibility that it might be causally related to reduced Mid-Holocene precipitation.

Although the impact of past fires on the structure and species composition of Holocene Amazonian forests is difficult to discern from pollen records, correlative peaks in charcoal and *Cecropia* and/or grass, spanning several millennia, in particular at Santa Maria, Geral and La Teta-2, point to burning of sufficient frequency to maintain forests in a continually disturbed, early successional state.

## 4. Conclusions and implications for the future

In the most seasonal, ecotonal regions of the Amazon lowlands, the drier climate of the Early–Mid-Holocene caused either the replacement of forest by savannah (Carajas) or supported the continued presence of savannahs in regions which were previously unforested (e.g. Chaplin, Loma Linda). On the eastern slopes of the Andes, reduced cloud cover caused replacement of cloud forest taxa by lowland tree taxa. Even in the wettest central part of the Amazon (Pata), closed-canopy forest may have given way to more open vegetation, consistent with Mid-Holocene drying.

Many sites show Early–Mid-Holocene peaks in *Cecropia* pollen, constituting clear evidence of disturbance. This disturbance may have been caused by drought, fire or humans, or a combination of all three, the probable cause at a given site depending on how well the palaeoclimate, pollen and charcoal patterns match one another, as well as the precipitation regime and fire return interval of the locality today. Even where it is clear that the disturbance was driven by fire (e.g. Santa Maria), the cause of fire may itself be an issue. Palaeo-Indians are the most probable source of ignition, even at seasonal sites such as Santa Maria, where the charcoal peak supports the hypothesis that a *Cecropia* phase spanning several millennia is best explained by Palaeo-Indians maintaining the forest in an early successional state using fire. In fact, the widespread evidence for these long-lasting *Cecropia* phases suggests that humans, rather than climate, may have been the key agents of disturbance of Holocene forests in many parts of the basin, especially if ‘pre-Conquest’ Amazonia was much more densely populated than previously thought (Erickson 2000; Heckenberger *et al.* 2003, 2007). However, a drier climate would have had an important influence by making forests more combustible. Anthropogenic burning would therefore have been a more effective tool for forest clearance and, through more frequent fire leakage, would have led to an increase in large wildfires as occurs today during particularly severe droughts (Aragão *et al.* 2007).

Our analysis shows that, notwithstanding floristic changes, the forest biome in most parts of Amazonia appears to have been remarkably resilient to climatic conditions significantly drier than those of today, despite widespread evidence of forest burning. Only in ecotonal areas did forests give way to savannahs (e.g. Carajas). Although the effects of continually rising CO_2_, and different climate change scenarios, upon Amazonia's forests over the twenty-first century remain uncertain (Cramer *et al.* 2004), our insights from the distant past suggest that the Amazon forest ‘dieback’ scenario simulated by Cox *et al.* (2000) and Betts *et al.* (2004) is unlikely. However, the absence of Holocene palaeotemperature records from lowland Amazonia means that the degree to which Early–Mid-Holocene Amazonian ecosystems can be considered an appropriate analogue for the future is uncertain. A projected temperature increase of 3°C over the twenty-first century (Malhi & Wright 2004), in combination with drying and forest fragmentation, would be expected to increase water stress and vulnerability to dieback, although this may be offset by higher CO_2_ concentrations. Of much greater cause for concern should be the unprecedented rates of deforestation (Laurance *et al.* 2001), forest fragmentation (Laurance *et al.* 1997, 2000) and uncontrolled burning (Cochrane *et al.* 1999; Nepstad *et al.* 1999), which are much more serious and immediate threats than climate change.

## Figures and Tables

**Table 1:** Site metadata.

site name	latitude	longitude	elevation (m)	country	local vegetation	ecoregion	investigator
*palaeoclimate records*							
Junin	−11.0000	−75.0000	5700	Peru		Peruvian Yungas	Seltzer *et al.* (2000)
Titicaca	−16.1344	−69.1553	3810	Bolivia/Peru		Central Andean wet puna	Baker *et al.* (2001)
Sajama ice cap	−18.1000	−68.8833	6542	Bolivia		Central Andean dry puna	Thompson *et al.* (1998)
*palaeovegetation records*							
lowland ecotonal sites							
Chaplin	−14.4667	−61.0667	200	Bolivia	humid rainforest	Madeira–Tapajós moist forests	Mayle *et al.* (2000) and Burbridge *et al.* (2004)
Bella Vista	−13.6167	−61.5500	190	Bolivia	humid rainforest	Madeira–Tapajós moist forests	Mayle *et al.* (2000) and Burbridge *et al.* (2004)
Carajas	−6.5833	−49.5000	750	Brazil	humid rainforest/savannah	Xingu–Tocantins–Araguaia moist forests	Absy *et al.* (1991) and Sifeddine *et al.* (2001)
Loma Linda	3.3000	−73.3833	310	Colombia	gallery forest/savannah	Apure–Villavicencio dry forests	Behling & Hooghiemstra (2000)
Chenevo	4.0833	−70.3500	150	Colombia	gallery forest/savannah	Negro–Branco moist forests	Berrio *et al.* (2002)
southern sites							
Parker	−12.1406	−69.0215	276	Peru	humid rainforest	Southwest Amazon moist forests	Bush *et al.* (2007*a*)
Gentry	−12.1773	−69.0977	258	Peru	humid rainforest	Southwest Amazon moist forests	Bush *et al.* (2007*a*)
eastern sites							
Saracuri	−1.6788	−53.5703	18	Brazil	humid rainforest	Uatuma–Trombetas moist forests	Bush *et al.* (2007*b*)
Santa Maria	−1.5783	−53.6054	17	Brazil	humid rainforest	Uatuma–Trombetas moist forests	Bush *et al.* (2007*b*)
Tapajos	−2.7758	−55.0828	15	Brazil	humid rainforest	Madeira–Tapajós moist forests	Irion *et al.* (2006)
Comprida	−1.6249	−53.9996	130	Brazil	humid rainforest	Uatuma–Trombetas moist forests	Bush *et al.* (2000)
Geral	−1.6469	−53.5955	130	Brazil	humid rainforest	Uatuma–Trombetas moist forests	Bush *et al.* (2000, 2007*b*)
Rio Curua	−1.7347	−51.4549	3	Brazil	*varzea*–*igapó* forest	Xingu–Tocantins–Araguaia moist forests	Behling & da Costa (2000)
western and central sites							
Pata	0.2667	−66.0667	300	Brazil	humid rainforest	Negro–Branco moist forests	Bush *et al*. (2002, 2004*a*)
Calado	−3.2667	−60.5833	23	Brazil	*varzea*–*igapó* forest	Japurá–Solimoes–Negro moist forests	Behling *et al.* (2001)
Maxus-4	−0.4500	−76.6166	3	Ecuador	humid rainforest	Napo moist forests	Weng *et al.* (2002)
Andean sites							
Chochos	−7.6363	−77.4746	3285	Peru	sub-paramo/sub-Andean forest	Peruvian Yungas	Bush *et al.* (2005)
La Teta-2	3.0833	−76.5333	1020	Colombia	disturbed forest	Cauca Valley dry forests	Berrio *et al.* (2002)
Surucucho	−3.0625	−78.0000	3180	Ecuador	sub-paramo/sub-Andean forest	Napo moist forests	Colinvaux *et al.* (1997)
Consuelo	−13.9500	−68.9833	1360	Peru	cloud forest	Bolivian Yungas	Bush *et al.* (2004*b*) and Urrego (2006)
